# Strategies for Controlling Blood Pressure Among Low-Income Populations in Georgia

**Published:** 2008-03-15

**Authors:** J. Nell Brownstein, Roberta Constantine, Sonja Hoover, Lashawn Wordlaw-Stinson, Diane Orenstein, Rosanne Farris, Patricia Jones

**Affiliations:** Centers for Disease Control and Prevention, Division for the Prevention of Heart Disease and Stroke; Research Triangle Institute, Waltham, Massachusetts; Research Triangle Institute, Waltham, Massachusetts; Research Triangle Institute, Waltham, Massachusetts; Division for the Prevention of Heart Disease and Stroke, National Center for Chronic Disease Prevention and Health Promotion, Centers for Disease Control and Prevention, Atlanta, Georgia; Division for the Prevention of Heart Disease and Stroke, National Center for Chronic Disease Prevention and Health Promotion, Centers for Disease Control and Prevention, Atlanta, Georgia; Stroke and Heart Attack Prevention Program, Georgia Department of Human Resources, Atlanta, Georgia

## Abstract

**Background:**

In Georgia an estimated 32% of blacks and 28% of whites have high blood pressure. In 2004 the rate of death from stroke in Georgia was 12% higher than the national average, and blacks in the state have a 1.4 times greater rate of death from stroke than that of whites.

**Context:**

The Georgia legislature funds the Stroke and Heart Attack Prevention Program (SHAPP) to provide treatment and medications for indigent Georgians. The median rate of blood pressure (BP) control among SHAPP enrollees is approximately 60%, compared with the national average of 35%.

**Methods:**

SHAPP was evaluated through interviews with key health care and administrative staff and through focus groups of patients in two clinics.

**Consequences:**

Outcomes for patients were increased knowledge of their BP and improved compliance with taking medication and keeping clinic appointments.

**Interpretation:**

Successful components of SHAPP include an easy enrollment process; affordable medication; use of evidence-based, documented protocols and patient tracking systems; routine follow-up of patients; and effective communication between staff and patients. Challenges and recommendations for improvement are identified.

## Background

In the United States high blood pressure (HBP), or hypertension (HTN), is of great concern because of its widespread prevalence and its impact on the incidence of heart disease and stroke (1). Nationally, nearly one-third of adults have HTN (mean systolic BP ≥140 mm Hg or mean diastolic BP ≥90 mm Hg, based on two measurements taken at least 2 minutes apart), and another 28% have prehypertension (BP of 120–139 mm Hg [systolic] and/or 80–89 mm Hg [diastolic]) (1). Nearly 70% of people with HTN do not have it under control (1,2). HTN is particularly common among blacks, who have a 30% higher prevalence than whites (3). Compared with whites, blacks develop HTN earlier in life, have higher average BP, and have higher rates of complications from HTN, such as stroke, heart attack, and end-stage renal disease (2,4-5). Prevalence of HTN is higher in the southeastern states than in other U.S. states and is higher among blacks than among whites (5). Lifestyle changes recommended for controlling HTN include maintaining a healthy weight, stopping smoking, maintaining a healthy diet, and engaging in regular physical activity (6). For some patients, antihypertensive medications may be indicated, and adherence to treatment is essential.

In Georgia an estimated 32% of blacks and 28% of whites have HTN (6,7). In 2004 the rate of death from stroke in Georgia was 12% higher than the national average (7), and the burden of stroke is especially heavy for the state's black population. Blacks in the state have a 1.4 times greater rate of death from stroke than that of whites (8); these disparities might be related to differences in risk factors among blacks and whites, lower socioeconomic and educational status among blacks, cultural dietary practices of blacks, and blacks' poor access to treatment for chronic disease (4,9). The trends in Georgia are similar to the trends in other southeastern states (9).

## Context

In 1974 the Georgia legislature founded the Stroke and Heart Attack Prevention Program (SHAPP), an education and direct-service program targeting low-income Georgians with HBP. To be eligible, patients must be 18 years of age or older, have proof of income not to exceed 200% of the current federal poverty guidelines, and have proof of having no prescription drug benefits (patients who need only education and monitoring do not have to meet eligibility requirements). More than 1.1 million Georgians, or more than 13% of the population, are below the federal poverty level, and poverty is associated with poor control of HBP, diabetes, and other chronic conditions (10).

SHAPP serves 16 of Georgia's 18 public health districts. Patient services, provided through county health departments and one independent contractor, include screenings, referral to doctors, diagnosis, treatment, and follow-up. Patients can refer themselves to SHAPP, or they can be referred by a physician. Treatment protocols are based on recommendations of the Joint National Committee on Prevention, Detection, Evaluation, and Treatment of High Blood Pressure (2). Through SHAPP, indigent adults with medical conditions are eligible for HTN medications at low or no cost and to have treatment outcomes monitored.

Physicians are available for consultation to people with complications or problems. The clinics use tracking systems for appointments and for monitoring patient status, and public health nurses, serving as case managers, monitor patients for problems, keeping appointments, and adherence to taking medications (11). SHAPP also provides patient, professional, and public education.

Although almost half a million people in Georgia could be eligible for SHAPP, fewer than 16,000 patients were served in 2003 (11). The number of patients served is limited because of funding restrictions. The largest population segments served include blacks and people aged 30–59 years. Rates of controlling HTN range from 38% to 84% among Georgia's public health districts (6); the median rate among Georgia public health districts is approximately 60% (11).

We report the outcome of an evaluation using interviews with SHAPP staff and focus groups with patients enrolled in SHAPP to determine the factors contributing to successful control of HTN. The study involved SHAPP in two clinics, one in Georgia's east central public health district and another in Georgia's coastal public health district. The data gathered through these qualitative methodologies proved to be a rich source of information and provided insights about the successful HTN control rates in the two clinics. A cost-effectiveness evaluation of the two clinics estimated enhanced patient health, decreased adverse events, and reduced costs to the state; these results are published elsewhere (11).

## Methods

### Selection of districts

We selected the east central and coastal public health districts for several reasons, including the districts' successful rates of BP control, sufficient cost data on patient and pharmaceuticals, use of two different patient tracking systems, and geographic and demographic diversity (9). In 2003 the east central district reported a BP control rate of 64%, and the coastal district reported 60%. The east central district served 543 SHAPP patients in 13 counties through 15 clinics, and the coastal district served 1632 SHAPP patients in 13 counties through 14 clinics (SHAPP annual performance report 2003, unpublished data). Clinic 1 (31 patients), in the east central district, and Clinic 2 (125 patients), in the coastal district, were selected for the focus groups.

### Interviews and focus groups

Four telephone interviews and one focus group were conducted in each district. Administrative staff, nurses, and physicians participated in telephone interviews. Clinic staff, who had established relationships with the patients and were able to facilitate the process, recruited patients (who had been in SHAPP for at least 6 months) for the focus groups. Each focus group had six patients: three with uncontrolled HBP (mean systolic BP ≥140 mm Hg or mean diastolic BP ≥90 mm Hg, based on two measurements taken at least 2 minutes apart) and three with controlled HBP. A gratuity ($15) was provided to participating patients; the focus groups lasted approximately 90 minutes.

Interviews with SHAPP administrators and clinic staff examined the program's challenges, patient access to the program, patient outcomes, and recommendations for improvement. The focus groups covered perceptions of the program, experiences with HBP, effects of participation, access to the program, and recommendations.

Institutional review board approval was obtained from the Centers for Disease Control and Prevention, Research Triangle Institute (RTI), and the Georgia Department of Human Resources. Before conducting the focus groups, informed consent was explained to participants, and the consent form was reviewed with the participants and signed.

Both the interviews and the focus groups were conducted by a two-person team from RTI, using the success case method for qualitative evaluation (12). The team identified subtopics for following up on potential responses, a process designed to ensure that all critical issues were addressed. The interviews and focus groups were conducted in February and March of 2005.

After each interview and each focus group, the research team held a debriefing session in which notes they had taken were integrated; this strategy provides context for understanding the responses and conceptualizes the data in terms of the study objectives. The themes were grouped and analyzed using the qualitative software ATLAS 5.0 (ATLAS.ti Scientific Software Development GmBH, Berlin, Germany).

## Consequences

### Staff interviews

SHAPP staff identified two key challenges with the program: 1) funding and staff levels, and 2) patient care and treatment. Key challenges and components of success identified by staff are summarized in the Table.

**Funding and staff**

Funding challenges posed the most immediate threat to keeping the program in operation. All of the staff emphasized the negative impact of state budget cuts on recruiting staff, enrolling patients, and obtaining HBP medications.

These funding cuts affected the hiring and retention of nursing staff. One staff member stated that 75% of newly hired nurses within all the health districts quit within 6 months for better pay or more reasonable caseloads elsewhere. The SHAPP caseload was high at any given time, because only two nurses managed SHAPP patients and coordinated SHAPP and other patients in each clinic. Despite the staff shortage, focus group participants described interactions with the clinic staff positively.

**Patient care and treatment**

The staff found working with the client population to modify their lifestyle behaviors (i.e., diet, physical activity, smoking, alcohol consumption) an ongoing challenge, because many patients were reluctant to significantly change their lifestyles.

Often the patients presented with comorbidities (e.g., diabetes). However, because SHAPP is uniquely designed to address primary HTN, patients who required other medications needed to seek care elsewhere for those conditions. Many patients did not have any other access to health care or funds for medications.

**Components of success**

The staff identified several components of SHAPP that were critical to patients' success in lowering BP:

*Staff commitment to patient care and belief in the value of SHAPP for the community.* The staff indicated that SHAPP fills a critical need for indigent community members.

*Effective communication between providers and patients.* SHAPP was successful in managing HTN, as judged by BP control rates. Control rates for SHAPP enrollees were 60% and 64% in the two districts, compared with a 35% control rate nationally. Increased control rates among SHAPP enrollees were accomplished through monitoring, counseling, and compliance with medication. Staff spoke with patients whose HTN was resistant to treatment to learn whether nonadherence to lifestyle recommendations, noncompliance with medications, or changes in medication dosage or drug type were the cause.

*Support for patients and families.* The nurses provided counseling and education, built skills, and recruited parents or caregivers to support patients in complying with treatment.

*Available and affordable medications.* SHAPP filled a need in the community by supplying most of the patients (90%–98%) with medications to help control their BP. The staff believed that patients would have difficulty in obtaining affordable medications elsewhere.

*Ongoing tracking and monitoring system (including system reminders) to maintain continuity of care*. Nurses provided counseling to emphasize the importance of keeping appointments. In one of the clinics, clients were telephoned the day before the appointment or, if the appointment was missed, later that day. The nurses followed up missed appointments with letters. The other clinic used a computerized tracking and monitoring system, which provided nurses with a computer printout of an active patient list so patients could be screened for attendance and BP control.

### Focus groups

Patients identified four program elements that were critical to success in BP control: ease of enrollment, staff commitment, affordable medications, and education about lifestyle modifications. Elements of SHAPP that patients identified as critical to their success in controlling HTN are summarized in the Table.

**Ease of enrollment**

Because knowledge of SHAPP was obtained generally by word of mouth, self-referral was as common as a referral from a physician or local health department. Participants believed that enrollment was easy. As one focus group participant stated: "It wasn't hard. I mean, you'd give them all the information they need." Participants also appreciated the staff's professionalism: "They didn't make you feel embarrassed or anything — that you were, you know, poverty level." The patients were only turned away if they were not income eligible or if they already had other insurance (i.e., Medicaid). However, ineligibility was rarely a problem, because the program was structured to target people not reached by the health care system.

**Staff commitment to and communication with patients**

Focus group participants clearly indicated that the caring SHAPP staff exceeded their expectations. They described the staff as "real nice, real good," "helpful," and even "if you don't lose but a pound, they just make you feel good." As one participant stated, "They're helpful in all ways and they make time for you." It was a significant benefit to the patients to have staff available to provide counsel and support for lifestyle modifications and compliance with appointments.

Participants mentioned that when the "appointment's maybe a little past," nurses tried to reschedule them or counseled them over the phone. When asked about being seen in the clinic, another participant responded: "Every 3 months, I think it is. That's the way it is with me. They set it up. Or if I need to come earlier or something . . . just call them and they'll know you, they'll probably set you up earlier."

**Availability of affordable medications**

Participants indicated the program had helped them immeasurably in being able to afford medications. One stated, ". . . [I]f it hadn't been for the program, I'd be dead right now . . . because I couldn't afford the medicine," and another said, "[I]f it wasn't for this program, we couldn't make it." According to the participants, SHAPP succeeded in its goal of providing affordable HBP medications. Before enrolling, many of the participants said they could not afford their BP medications and other medications. However, after enrolling the participants were all able to afford their BP medications and expressed thanks for the program.

**Education about lifestyle modifications**

Focus group participants and staff who were interviewed generally agreed that the program was effective in lowering BP. Focus group participants indicated that it was an ongoing effort to learn how to control their BP but that SHAPP gave them the tools they needed. When asked what they had learned through SHAPP that they did not learn from their physicians, one respondent answered, "To try to control [my BP] . . . well, eat the right foods. . . . The medication helps a lot, too."

## Interpretation

Overall, the clinics confronted the same challenges in keeping SHAPP in operation and successful. From the interviews, we found that the most common patient outcomes were improvements in knowledge of HTN, compliance with medications, and keeping appointments. This is consistent with outcomes of other community-based studies (9,13).

Through the interviews and focus groups, four themes emerged that explained much of the success of the two clinics: program access, staff dedication, continuity of care, and patient satisfaction. The patients found that the staff made time for them, were accepting and nonjudgmental of their low-income status, and treated them well. The staff were dedicated and consistently voiced the opinion that SHAPP filled a need in the community; they understood the importance of their work and that the patients would be unlikely to receive care otherwise. The patients also expressed this theme; without SHAPP their HTN would not be addressed. The satisfaction expressed by the patients suggests that their expectations of care and requests for medicine were met and that they trusted the staff. Their high level of adherence to HBP treatment reflected their satisfaction.

Both clinics worked with the patient population to modify lifestyle behaviors, which was an ongoing challenge. Interestingly, in another qualitative study of 93 blacks (mostly low-income and female) with HTN, many of the patients believed that if they took their medications as prescribed they would not have to adopt healthier lifestyles (14). Some SHAPP patients may have shared this belief, which might help to explain the source of the nurses' frustrations with the lack of significant lifestyle modifications in the patient population.

Many of the critical components for success identified in this case study also were found in population-based randomized controlled trials (on HTN) and other BP control studies testing community health workers (CHWs) as members of health care delivery teams. Many of these studies developed strong community partnerships, provided medications, and, through nurse-supervised CHWs, provided education, counseling, skill building, and continuity of care through monitoring and tracking, including appointment reminders (13). Other nurse-based HTN interventions also have resulted in successful BP outcomes (13,15,16). SHAPP nurses provided continuing education based on the traditional medical model that HTN is a chronic, largely asymptomatic, incurable condition that requires lifelong compliance with treatment (2). The HTN control rate of the two clinics would indicate that most of the patients understood this model. SHAPP is based on the Chronic Care Model, which involves patients in their own care (17). Thus, these high-performing clinics have translated, adopted, and implemented evidence-based research findings into everyday practice. One strategy that might aid the nurses in SHAPP programs is to recruit, train, and integrate CHWs into the care team. Supervised by nurses, CHWs can provide health education, counseling, and tracking of patients (13), reducing the burden on nurses and possibly allowing for more participants in SHAPP.

The staff offered several ideas for improving SHAPP in the areas of outreach and primary prevention:

*Outreach*. Link SHAPP patients and the community at large to resources to support healthy lifestyle habits, identify at-risk people, and acquire a mobile clinic unit.

*Prevention*. Ideas included the following: 1) work collaboratively with other state-supported programs for prevention and treatment; 2) enhance or strengthen existing services (examples included adding more nursing staff and an on-site physician and hiring at competitive salaries, particularly nurse practitioners for their specialized training); 3) provide nursing staff with specialized training in cardiovascular medicine; and 4) expand SHAPP funding and staff to meet the challenges of patients with comorbid conditions. If patients are given coordinated treatment for health problems related to cardiovascular disease, the results will include improved patient health and more cost-effective operations.

*Sustainability*. Provide stable legislatively proportionate funding so adequate staff can be recruited and retained. The staff were passionate in their views that SHAPP fulfilled a need in the community and the community would witness an increase in uncontrolled HTN, an increase in hospitalizations from complications of HBP, and, within a 5-year period, an increase in overall mortality rates from heart attack if SHAPP was discontinued. The staff expressed concern that without SHAPP, patients would not self-manage their HBP.

Additionally, given the estimated cost savings, improved patient outcomes, and fewer expected adverse events, we suggest that other states should consider and test the promising practice demonstrated by SHAPP (8). If indigent people do not receive education and services for treatment of their HTN, adverse events, higher rates of death, and overwhelming medical and societal costs will increase substantially. An earnest and ongoing commitment by the federal and state governments and others is required to provide sustainable funding and adequate manpower to provide HTN education and services.

Our study had a few limitations. We examined SHAPP in only two clinics, because conducting a case study of most or even all of the clinics would have been too costly. The two clinics we studied were not selected randomly, and the results are not generalizable nationally or within the state. These limitations notwithstanding, exploring promising programs in the field will provide insight into what works for patients and will help to move public health practice forward.

## Figures and Tables

**Table.** Results From Staff Telephone Interviews and Patient Focus Groups, Stroke and Heart Attack Prevention Program (SHAPP), Georgia, United States, 2005

**Components of Program Success**	
**Staff Comments**	**Patient Comments**
Staff commitment to patient care	Ease of enrollment
Belief in value of SHAPP for community	Staff commitment to and communication with patients
Effective communication between providers and patients	Availability of affordable medications
Support for patients and families	Education about lifestyle modifications
Available and affordable medications	
Ongoing patient tracking and monitoring system to maintain continuity of care	

**Program Challenges^a^**	

Funding and staff	
Patient care and treatment	

a Comments from staff telephone interviews only.
